# Bedsteads

**Published:** 1892-12-17

**Authors:** 


### PRACTICAL DEPARTMENTS.

BEDSTEADS—(*continued.*)

The height of bedsteads must be regulated by various considerations, and these naturally differ considerably in many institutions. Where the inmates get up and move about during any part of the twenty-four hours their beds are looked upon, very properly, only as places to sleep in, and therefore so long as they are not inconveniently low, or awkwardly high, the exact elevation is of small importance.

However, there are, unfortunately, in hospitals, and in some other institutions, a great many patients who will have to live in bed during nearly the whole time they reside within the walls, and for such as these the altitude of the iron frame on which their mattress is placed becomes of considerable importance to themselves and to their attendants. Very low bedsteads are never to be desired for several good reasons. It is very difficult to keep the floor beneath properly swept when the legs are so short as to give small space for broom or for daylight to penetrate, and therefore a low bed is liable to be a far from hygienic one. It is also a needlessly wearying experience for a nurse who is constantly obliged to stoop in a fashion very exhausting to herself, whilst the patient is in no wise benefited by her discomfort. The doctors, too, strongly condemn very low beds for both these reasons, and also as an unnecessary discomfort to themselves personally. On the contrary, a particularly high bedstead is not altogether to be commended; it may be pleasant enough for the patient, who is, as we said before, a prisoner thereon, but when he is allowed to get in and out of it once more he will suffer fatigue from having to mount too high. So a moderately, but not an excessively, high bedstead is perhaps the ideal one. To have plenty of light and to be able to see through the windows, these are no mean advantages. There may not be much of a view, but any glimpse of life outside the four walls of a sick-room is a boon to most sick persons. For example, at the Sussex County Hospital a special elevation secures to the patient an excellent prospect, and to be able to look away at the sea and to command a wide extent of sky are matters of considerable importance to the bedridden, whether their confinement is for a short or a long term of days. We have seen many excellent forms of spring mattresses to suit every taste, but in making a selection strength and simplicity are the two points to be specially taken into account. Strength and good workmanship provide that security which is as essential for a patient's comfort as the elasticity or spring on which so much store is set. Simplicity we mention particularly with regard to the after use of the structure. This should be easy to fix and unfix, and to mend also, should a necessity arise for repairs, and above all things, a bedstead should not be difficult to keep clean. If this is essential in private houses, how much more is it to be insisted on in institutions, and, of course, amongst the sick it is impossible to dwell too strongly on the banishment of dusty accumulations. When a hospital bed has been carbolised it ought to be a matter of impossibility for any corners or intricacies of design to combine the evils of a dust-trap with any supposed decorative art. All these points may seem of small importance; but it is just this careful attention to details which makes furniture of all kinds sanitary or the reverse.

The institution bedstead made by Messrs. Stidolph, of Dartford, has much to commend it in the way of cleanliness, for the bottom is made of strong canvas or sacking, so tense as to possess many of the advantages of wire spring beds. This canvas is double, like a roller towel, and so efficiently and evenly strained that it really forms a most satisfactory, firm, yet buoyant, resting place. Of course, the frequent washing of the sacking is a simple and inexpensive matter. A bed-rest on the same roller principle is a very satisfactory affair, and it is absolutely immovable and indestructible. The cloth is strained over bars of iron, which form a simple and effective framework; when the sacking is fixed by webbing straps the result is most practically satisfactory. The rest can be fitted very speedily to a bedstead, and one key is designed to fulfil three or four different purposes, which is naturally a wise arrangement. Stidolph's catalogue shows well the different points to which we have alluded.

